# Editorial: Applications of mindfulness in media and communication studies

**DOI:** 10.3389/fpsyg.2026.1886419

**Published:** 2026-06-26

**Authors:** Hao Chen, Si-Qi Zeng, Ye-Hui Tu, Chao Liu

**Affiliations:** 1 School of Film Television and Communication, Xiamen University of Technology, Xiamen, China; 2 School of Journalism and Communication, Hua Qiao University, Xiamen, China

**Keywords:** digital learning, ethical communication, media literacy, mindful media consumption, mindfulness, mindfulness-based interventions

In today's media environment, individuals navigate an always-on stream of algorithmically curated information, social comparison cues, and emerging AI-mediated interactions. These mediated conditions can amplify cognitive overload, emotional reactivity, fragmented attention, and interpersonal strain. Within this context, mindfulness has become increasingly relevant—not only as an intrapersonal practice, but also as a communication-related resource that shapes how people attend to, interpret, and respond to mediated experiences.

This Research Topic, “*Applications of mindfulness in media and communication studies*,” brings together 21 articles that examine mindfulness across social media use, news exposure, online learning, AI adoption, digital self-regulation, prosocial responses to media content, and immersive technologies such as virtual reality. Collectively, the contributions highlight mindfulness as a key mechanism for understanding and improving human functioning in the contemporary communication ecology.

## 1 The evolution of mindfulness in media and communication contexts

Mindfulness has traditionally been studied as a psychological capacity centered on present-moment awareness and non-judgmental attention. In the digital age, however, mindfulness is increasingly being reframed as a media-facing self-regulation capacity: it can buffer against technology-related stressors, reshape media consumption patterns, and support healthier engagement in online social life.

The studies in this Research Topic reflect several methodological and conceptual shifts. First, mindfulness is increasingly modeled as a moderator that dampens harmful media effects (e.g., FOMO, fatigue, dependence-related sleep impairment). Second, it is also examined as a mediator that channels cognitive and emotional processes toward adaptive outcomes (e.g., career decision-making efficacy, perceived support via improved relationships). Third, the field is expanding toward technology-forward contexts, including AI adoption and VR-based training, as well as toward ethics and social harmony, where mindful media engagement becomes relevant to tolerance, peace, and responsible news use.

## 2 Articles in this Research Topic

To provide a coherent overview, the 21 articles in this Research Topic can be understood as clustering into five complementary lines of work: (1) conceptual and normative reframings of mindfulness in media and communication, (2) digital media use, self-regulation, and everyday wellbeing, (3) media education, AI, and youth development, (4) relational, prosocial, and ethical communication, and (5) technology-mediated interventions, delivery formats, and emerging methodological directions.

### 2.1 Conceptual and normative reframings of mindfulness in media and communication

“*Framing the mind: media, metaphors, and mindfulness*” Meaden offers a conceptual contribution on how media metaphors shape public understandings of mindfulness. By treating framing as a cognitive bridge between media discourse and contemplative practice, this article provides a valuable theoretical entry point for communication scholars seeking to examine mindfulness as a culturally mediated construct (Meaden).

“*Reconsidering intrapersonal communication through an interdisciplinary lens*” Bainbridge et al. revisits intrapersonal communication with broader theoretical tools. This contribution is important because it helps position mindfulness not merely as a private psychological state, but as a communicative process within the self that is continually shaped by attention, interpretation, and reflection in mediated environments (Bainbridge et al.).

“*How should we approach negative news in the media? A ‘mindful' and ‘harmonious' consumption of negative news by users might be an answer*” Shabahang and Weber proposes a normative approach to negative news consumption. This article is especially timely because chronic exposure to distressing information has become a defining condition of contemporary media life, making mindful and balanced engagement an increasingly relevant communicative practice (Shabahang and Weber).

“*Factor analysis, emotional experience and behavioral feedback of contemporary Chinese youth participating in short-term monasticism: a qualitative study based on the online question-and-answer community Zhihu*” *Liu et al*. provides qualitative evidence on contemplative participation and meaning-making in an online community. This study enriches the Topic by showing how digital discussion spaces can host reflective narratives, experiential accounts, and culturally situated interpretations of contemplative practice (Liu et al.).

### 2.2 Digital media use, self-regulation, and everyday wellbeing

“*Mindfulness in social media exposure: the pressure-reducing valve for fear of missing out and social media fatigue*” Huang examines mindfulness as a protective factor in platform-based life. This study is important because it clarifies how mindful awareness may reduce pressure and fatigue generated by persistent social media exposure under conditions of continuous connectivity (Huang).

“*The role of FOMO and mindfulness in the relationship between mobile phone dependence and sleep quality among Chinese youth: a mediating and moderating analysis*” *Xie and Li* models the joint roles of FOMO and mindfulness in dependence–sleep pathways. Such evidence is valuable because it clarifies both the affective mechanisms involved and the buffering role mindfulness may play in reducing downstream harms associated with mobile phone dependence (Xie and Li).

“*The impact of multidimensional excessive social media use on academic performance: the moderating role of mindfulness*” Ma et al. addresses academic functioning under conditions of heavy social media engagement. This contribution is significant because it suggests that mindfulness may reduce the academic costs of excessive media use by strengthening attentional regulation and self-control (Ma et al.).

“*Mindfulness cognitive-based therapy combined with metaphor therapy can improve problematic social media use*” Xiong et al. targets problematic social media use through an intervention approach. This study extends the field beyond correlational inquiry by showing how mindfulness-informed treatment strategies may be translated into more applied responses to digital overuse (Xiong et al.).

“*Hoarding knowledge or hoarding stress? Investigating the link between digital hoarding and cognitive failures among Chinese college students*” Liu and Liu examines digital hoarding as a psychological and cognitive phenomenon. This work is important because it broadens the self-regulation agenda of the Topic, showing how accumulation behaviors in digital environments may be linked to cognitive strain and everyday failures (Liu and Liu).

“*Fostering life hope in urban green spaces through brief online mindfulness: findings from four studies with park visitors*” Luo and Zhang combines online mindfulness with environmental experience in everyday settings. This contribution is especially useful because it demonstrates how brief digital delivery may foster hope and positive psychological outcomes outside conventional therapeutic contexts, pointing to scalable forms of wellbeing support (Luo and Zhang).

### 2.3 Media education, AI, and youth development

“*Growth-oriented thinking and career decision-making self-efficacy among university students: the chain mediation role of mindfulness and future time perspective*” *Li et al*. positions mindfulness within developmental and educational communication contexts. This study is important because it shows how mindfulness may operate within a chain process linking growth-oriented thinking, future-oriented cognition, and career decision-making self-efficacy among university students (Li et al.).

“*Mindfulness and AI adoption: extending the technology acceptance model for Chinese media students*” *Lan, Liu, Chen et al*. integrates mindfulness into technology acceptance processes. This article advances TAM-based inquiry by suggesting that mindfulness may function as a psychological resource supporting more adaptive responses to AI-related opportunities and uncertainties in media education (Lan, Liu, Chen et al.).

“*How mindfulness shapes AI competence: a structural equation modeling analysis of mindfulness, AI literacy and behavioral intention in Chinese media students*” Lan, Liu, Xia further develops this line by modeling the formation of AI competence. This study strengthens the bridge between contemplative constructs and AI-era media education by showing how mindfulness may be associated with AI literacy and behavioral intention (Lan, Liu, Xia).

“*Mindfulness's moderating role applied on online SEL education*” Ho et al. connects mindfulness to online social-emotional learning. This contribution is significant because it highlights how contemplative capacities may shape the effectiveness of mediated pedagogy in increasingly digital learning environments (Ho et al.).

“*Shaping decision-making with screen time: video-based dialectical behavior therapy skills training for college students*” Mejía and Avila-Chauvet examines video-based DBT skills training among college students. Although not framed exclusively as mindfulness training, this study contributes to the broader conversation on digital mental health education by showing how screen-based instructional formats may support decision-making and self-regulatory skills (Mejía and Avila-Chauvet).

### 2.4 Relational, prosocial, and ethical communication

“*Reperceiving depression: how trait mindfulness enhances perceived support through improved doctor–patient relationships and stigma alleviation in depressed young adults*” Zhu et al. extends mindfulness into the domain of health communication. By emphasizing doctor–patient relational pathways and stigma reduction, the study illustrates how mindfulness may facilitate supportive communication and more constructive interpretations of mental health experiences (Zhu et al.).

“*Mindfulness and media-driven prosociality: effects of trait and state mindfulness on responses to conflict photojournalism*” Cheng et al. connects mindfulness to the moral-emotional processing of conflict imagery. This contribution is especially valuable because it clarifies when and how mindful awareness may shape prosocial responses to mediated suffering, thereby enriching media psychology research on empathy and moral response (Cheng et al.).

“*The impact of social media use on tolerance, community peace, online ethical awareness among adolescents in the United Arab Emirates*” Al Ketbi et al. foregrounds ethical and societal outcomes in digital communication. This study is important because it links media use to broader civic and moral concerns, extending the Topic from individual wellbeing to questions of tolerance, coexistence, and online ethical awareness (Al Ketbi et al.).

### 2.5 Technology-mediated interventions, delivery formats, and emerging methodological directions

“*Effectiveness of a mindfulness-based program with virtual reality to increase safe behaviors in workers of a mining company*” Guzmán et al. demonstrates VR-supported mindfulness in an occupational safety context. This work is important because it highlights the potential of immersive technologies to support behavioral change in high-risk environments where attentional control and safe conduct are especially consequential (Guzmán et al.).

“*Virtual reality vs. imagery: comparing approaches in guided meditation*” Jo et al. directly compares delivery modalities for guided meditation. Such comparisons are valuable because they clarify when technological immersion may offer benefits beyond traditional imagery-based practice, thereby informing future decisions about design, accessibility, and intervention fit (Jo et al.).

“*Effectiveness of mobile mindfulness training on stress, burnout, and work engagement of office workers: protocol for a randomized controlled trial*” Lee et al. contributes methodological groundwork for higher-quality evidence. By specifying a trial protocol targeting stress, burnout, and work engagement, this article supports stronger causal inference and a more cumulative research agenda in digital mindfulness studies (Lee et al.).

## 3 The significance of mindfulness in the digital media age

Taken together, the contributions in this Research Topic suggest that mindfulness has become a highly relevant construct for media and communication research in at least three important ways. First, mindfulness functions as a buffer against the psychological and behavioral strains of platform-based life, including fear of missing out, social media fatigue, dependence-related outcomes, and academic disruption. Second, mindfulness is increasingly being incorporated into technology-oriented frameworks, particularly in relation to AI adoption, AI literacy, and AI competence, underscoring its growing relevance in emerging communication environments. Third, mindfulness helps connect individual self-regulation to broader relational, societal, and ethical concerns by offering a useful lens for understanding prosociality, tolerance, peace, and more reflective engagement with negative news.

Collectively, this Research Topic shows that mindfulness is not merely a wellbeing technique or personal coping strategy. Rather, it is a communication-relevant construct that shapes how individuals attend to media, interpret mediated content, regulate digital habits, and engage with others under conditions of constant connectivity.

## 4 Future directions

Building on the evidence and ideas assembled in this Research Topic, future research may benefit from several directions: longitudinal designs to test durability of effects; cross-cultural comparisons to clarify cultural boundary conditions; mixed-methods approaches to connect mechanisms with lived experience; and design-oriented research examining how platform features and algorithmic architectures can support (or undermine) mindful engagement.

## 5 Conclusion

As we conclude this Research Topic on “*Applications of mindfulness in media and communication studies*,” we extend our sincere gratitude to all authors, reviewers, and editors who contributed to this Research Topic. The research presented here highlights mindfulness as a timely and versatile framework for understanding mediated life—spanning mental health, education, technology adoption, ethics, and immersive intervention design.

In an age where media environments increasingly shape attention, emotion, and social relationships, mindfulness offers more than individual relief; it provides a pathway toward more reflective, balanced, and responsible engagement with digital communication.

